# How Does the Driver’s Perception Reaction Time Affect the Performances of Crash Surrogate Measures?

**DOI:** 10.1371/journal.pone.0138617

**Published:** 2015-09-23

**Authors:** Yan Kuang, Xiaobo Qu, Jinxian Weng, Amir Etemad-Shahidi

**Affiliations:** 1 Griffith School of Engineering, Griffith University, Gold Coast, Australia; 2 School of Management, Shanghai University, Shanghai, China; 3 College of Transport and Communications, Shanghai Maritime University, Shanghai, China; University of California Berkeley, UNITED STATES

## Abstract

With the merit on representing traffic conflict through examining the crash mechanism and causality proactively, crash surrogate measures have long been proposed and applied to evaluate the traffic safety. However, the driver’s Perception-Reaction Time (PRT), an important variable in crash mechanism, has not been considered widely into surrogate measures. In this regard, it is important to know how the PRT affects the performances of surrogate indicators. To this end, three widely used surrogate measures are firstly modified by involving the PRT into their crash mechanisms. Then, in order to examine the difference caused by the PRT, a comparative study is carried out on a freeway section of the Pacific Motorway, Australia. This result suggests that the surrogate indicators’ performances in representing rear-end crash risks are improved with the incorporating of the PRT for the investigated section.

## Introduction

The increase in motor-vehicle crash has been well recognised as a major health problem by World Health Organization (WHO). It is stated that around 1.24 million people lost their lives and 50 million were injured in crashes on the roads around the world each year. Further, as the leading cause of death for young people aged 15–29 years, road crashes take an enormous toll on individuals and communities as well as on national economies [1]. In Australia, it was reported that the social cost of vehicle crashes was estimated as AUD 27 billions per annum with devastating social impacts [2]. Among these crashes, those on motorways are recognized as more severe than crashes on urban streets in terms of their consequences. According to the crash data provided by Department of Transport and Main Roads (DTMR) of Queensland, there are over 70% fatal crashes occurred on rural and inter-city roads each year. Inter-city motorways are usually designed to carry the travel demands among cities with high speed. Crashes occurred on motorways would potentially cause significant traffic delay and health, economic and environmental problems. In this regard, it is of great importance to investigate the traffic safety on motorways. The road safety has become a high-priority issue to traffic engineers and traffic authorities for decades. Researchers and engineers proposed many methods to improve road safety such as: the application of Intelligent Transportation System (ITS) programs [3, 4], the synergy of traffic energy saving [5–7] and autonomous vehicles [8, 9].

In order to reduce the crashes, many researchers have been contributed to find the possible reasons related to the crashes. Traditionally, a range of safety-related concerns are addressed by establishing the relationship between discrete crash counts and traffic/geometric parameters [10–13], relying heavily on historical crash data and statistical techniques [14–16]. However, as pointed out by Chin and Quek [17] and Tarko et al. [18], these traditional prediction models have some drawbacks and restrictions. First, due to the infrequence and sporadic occurrence of accidents, significant efforts are consumed on collecting and maintaining the appropriate data. Second, accidents are not always uniformly reported, which can produce biased conclusions. Third, these prediction models are purely dependent on statistical techniques and historical crash data, without taking into account the crash mechanism. Further, crash records for safety analysis are considered as a reactive approach, which requires a sufficiently large number of serious accidents to take place in advance. Consequently, surrogate indicators are proposed as a supplementary method of the accumulation of crashes in safety evaluation.

Surrogate indicators are firstly proposed and used to evaluate the treatment beforehand in medical sciences, and then utilized to reduce or eliminate the crashes by traffic engineers and researchers [19–28]. As suggested by Tarko et al. [18], surrogate events should satisfy two basic requirements: 1) surrogate events should be exacted from observable non-crash events by using some practical method (surrogate measure); 2) it is feasible to examine the relationship between these surrogate events and corresponding crash frequency and severity. Crash surrogate indicators have been well recognized as good safety indicators for analysing and predicting crashes. Firstly, surrogate events occur much more frequently than crashes with strong probabilistic properties. Secondly, as states between safe and crash, surrogate events can reflect the potential crash causality and mechanism. Last but not least, surrogate indicator is regarded as a proactive rather than reactive approach, which can proactively assess safety before crashes occur.

Although many surrogate indicators are proposed and applied to traffic safety during the past half century, to the best of our knowledge, little if not none takes into account the driver’s perception-reaction time (PRT). The primary objective of this study is to examine whether or not the incorporation of the PRT could improve the performance of a surrogate indicator. To this end, we firstly propose the modified surrogate indicators by taking into account the PRT. Based on the collected trajectory data on Pacific motorways, we validate the VISSIM simulation model by the error tests and trajectory comparison. Lastly, we evaluate the performances of the modified surrogate indicators based on the crash data on the motorway.

## Literature Review

Various surrogate indicators, including Time To Collision (TTC), Deceleration Rate To avoid Crash (DRAC), Crash Potential Index (CPI) and Proportion of Stopping Distance (PSD), are proposed and applied in safety evaluations. Based on the assumption that both vehicles keep the speeds unchanged during the process, the surrogate indicator TTC is defined as the time remains until a collision between two vehicles would have occurred [19, 20, 28], mathematically,

$$TTC=\{\begin{array}{cc} \frac{D_{1-2}}{V_{2}-V_{1}}, & \;\text{if}\;V_{2}>V_{1}\\ \;\infty \;, & \;\text{otherwise} \end{array}$$ (1)

where *D*_1−2_ represents the distance gap between the leading and following vehicle; *V*_1_ and *V*_2_ denote the speeds of the leading and following vehicles at the initial time, respectively. TTC has become one of the most well-recognized microscopic safety indicators, and been widely applied to evaluate the level of safety in different situations of traffic [27–31]. Further, Minderhoud and Bovy [32] develop the extended time to collision as the measures for traffic safety assessment based on TTC notion which can evaluate the risk more comprehensively by taking into account the full course of vehicles over space and time [20].

DRAC is another widely-used surrogate indicator. It is defined [20, 33] as the minimum deceleration rate required by the following vehicle to avoid a crash with the leading vehicle if the speed of leading vehicle is unchanged during the process. Mathematically, DRAC can be denoted as:

$$DRAC=\{\begin{array}{l} \frac{{\left(V_{2}-V_{1}\right)}^{2}}{D_{1-2}},\;\text{if}\;V_{2}>V_{1}\\ \;0,\;\text{otherwise} \end{array}$$ (2)

DRAC is recognized as an effective measure of safety performance in safety evaluation [32, 34]. The AASHTO [35] suggests that a given vehicle is in conflict if its DRAC exceeds a threshold 3.4 m/s^2^. Higher value of DRAC indicates a more dangerous car-following scenario.

CPI is defined [20, 36] as the aggregated probability for those car-following sceanrios where the following vehicles’ DRAC values exceed their braking capacities or Maximum Available Deceleration Rates (MADR) during a given time period, mathematically,

$$CPI_{i}=\frac{\sum_{t=0}^{N}P(DRAC_{i}(t)>MADR_{i})\cdot \Delta t}{T}$$ (3)

where *DRAC*_*i*_(*t*) and *MADR*_*i*_ are the DRAC and MADR value for the following vehicle of *i* ^*th*^ car-following scenario at discrete time *t* respectively; *N* and Δ*t* are the total number and the duration of time interval inspected; *T* is the total time duration investigated, where *T* = *N* ⋅ Δ*t*. MADR is vehicle and scenario-specific, and usually represented by truncated normal distributions [36, 37]. The surrogate indicator CPI is broadly used to evaluate the road risk in safety analysis [34, 37]. By taking into account the deceleration capacity of vehicles, CPI can deliver more comprehensive results due to the MADR distribution.

PSD is defined [20, 26, 34, 38] as the ratio between the remaining distance RD and the minimum acceptable stopping distance MSD, mathematically,

$$PSD=\frac{RD}{MSD}$$ (4)

where the remaining distance RD denotes the distance between the initial point and the potential point of collision, while the minimum acceptable stopping distance MSD represents the minimum stopping distance required based on the assumption of maximum deceleration rate used. PSD is measured by comparing the available and minimum acceptable stopping distances, all scenarios with PSD less than 1 are regarded as unsafe, where the collisions cannot be avoided with maximum acceptable deceleration rate taken. PSD is regarded as a good surrogate indicator and has been used for safety evaluation [34, 39].

Although the selected surrogate indicators are widely used in traffic safety evaluation, none of them takes into account the PRT. The PRT, which is defined as the minimum time required for the driver to react, is an important parameter in traffic safety and designing. For example, the National Association of Australian State Road Authorities (NAASRA) is currently using the PRT as the standard in the area of geometric road design for the visibility. Besides, it is used to estimate the stopping distance in the computation of horizontal and vertical profiles in highway design [40]. Further, the PRT also plays a significant role in the designing of the duration of yellow phase at signalized intersections [41]. During the onset of yellow phase, either a driver stops safely before the stop line or proceeds through the intersection before the end of yellow phase are both highly related to the PRT. In reality, the safety of intersections is maintained by alleviating the dilemma zone which is calculated based on the estimation of the PRT. Accordingly, PRT is of significant importance in traffic safety. However, this important parameter is not considered into the crash mechanisms of most widely used surrogate indicators. The possible reason would be the time gap between the study of surrogate indicators and PRT. It is found that most of the well-recognized surrogate indicators were proposed in 70’s of 19^th^ century, while most of the studies of PRT were carried out in this century. Before the distributions of PRT were obtained, the surrogate indicators have been proposed and widely used in safety evaluations. Hence seldom research has been done to establish the link between the PRT and surrogate indicators. Due to the ignorance of the PRT in most surrogate indicators, it is of great importance to take into account the PRT in safety evaluation due to its significance in crash mechanism. Wang and Stamatiadis [42–44] proposed a series of pioneering works to creatively incorporate the impact of the PRT in order to better evaluate intersection safety. Yet little research has been done for proactive motorway safety evaluation with the consideration of the PRT.

## Three Modified Surrogate Indicators

### Modified Deceleration Rate to Avoid a Crash (MDRAC)

This paper aims to examine whether the consideration of the PRT can improve the surrogate indicator’s performance or not. To this end, we reanalyse the crash mechanisms of selected surrogate indicators by considering the phase of the PRT.

**Fig 1:**
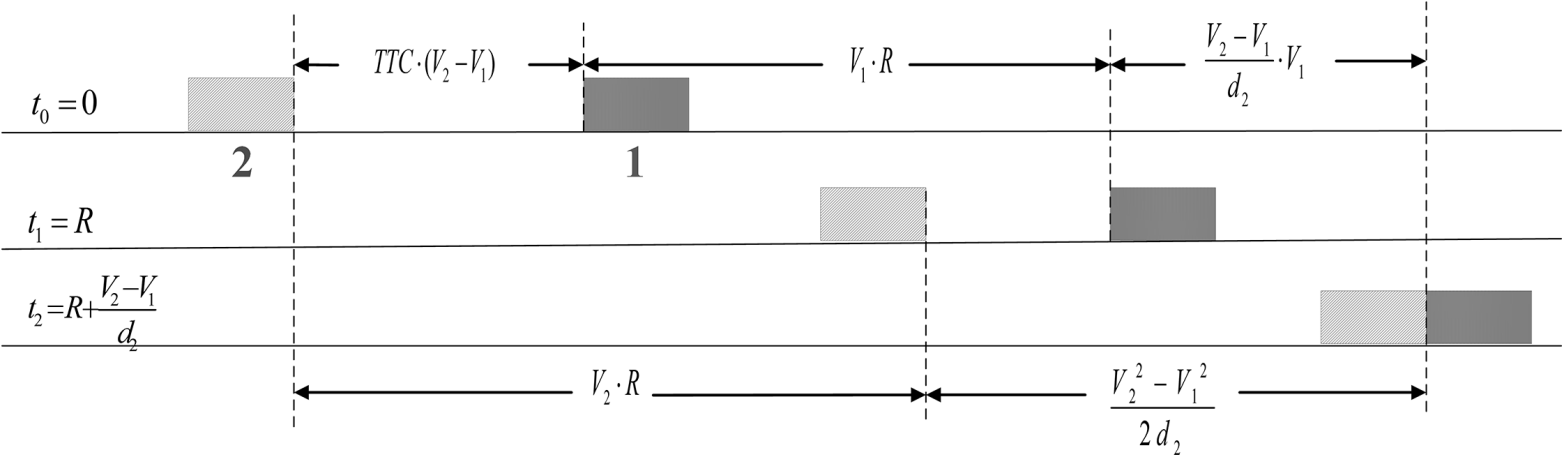
Conflict process of MDARC.

Fig 1 shows the crash mechanism of DRAC by taking into account the PRT, where a critical situation is depicted when the following vehicle just adapts its speed to that of the leading vehicle in time. As can be seen in Fig 1, the distance travelled by the following vehicle should be equal to the available distance, mathematically:

$$TTC\cdot (V_{2}-V_{1})+V_{1}\cdot R+\frac{V_{2}-V_{1}}{d_{2}}\cdot V_{1}=V_{2}\cdot R+\frac{V_{2}^{2}-V_{1}^{2}}{2d_{2}}$$ (5)

by simplifying Eq 5, MDRAC can be represented as:

$$d_{2}=\frac{V_{2}-V_{1}}{2(TTC-R)}$$ (6)

Accordingly, MDRAC can be expressed by speeds, PRT and TTC as follows,

$$MDRAC=\{\begin{array}{l} \frac{V_{2}-V_{1}}{2(TTC-R)},\;\text{if}\;TTC>R\\ \;\infty \;,\;\text{otherwise} \end{array}\;\forall V_{2}>V_{1}$$ (7)

where *V*_2_ and *V*_1_ represent the speeds of the following and leading vehicles, respectively; *R* denotes the PRT; *d*_2_ is the deceleration rate of the following vehicle; and TTC represents the time to collision value for the initial state (*t* = 0). This finding is also derived by Wang and Stamatiadis [42–44].

By comparing with DRAC, MDRAC is able to reflect the severity on the basis of TTC. For the same car-following scenario, the MDRAC can be varied due to the different PRTs of distinct drivers. If TTC is less than PRT, the following driver would not have enough time to react, a collision is not avoidable. In this paper, 3.4 m/s^2^ is suggested as the threshold of MDRAC by AASHTO [35].

### Modified Crash Potential Index (MCPI)

Since CPI describes the probability that a given vehicle DRAC exceeds its maximum available deceleration rate (MADR) or braking capacity, by using MDRAC instead of DRAC, the modified CPI (*MCPI*) can be represented as:

$$MCPI_{i}=\frac{\sum_{t=0}^{N}P(MDRAC_{i}(t)>MADR_{i})\cdot \Delta t}{T}$$ (8)

where *MDRAC*_*i*_(*t*) is the MDRAC value for the *i* ^*th*^ car-following scenario at discrete time *t*, estimated by Eq 7, *N* and Δ*t* are the total number and duration of time interval inspected; *T* is the total time duration investigated. According to the distribution of MADR, MCPI is measured based on the results of MDRAC. *MCPI* represents the crash potential index based on the consideration of the PRT, a higher MCPI indicates a more dangerous scenario.

### Modified Proportion of Stopping Distance (MPSD)

By taking into account the PRT, we propose modified surrogate indicator PSD by updating the minimum acceptable stopping distance (MSD). In this conflict process, the modified MSD (MMSD) should contain two parts: 1) the distance travelled for the following vehicle during its PRT; 2) the braking distance travelled by the following vehicle its PRT till it stops, mathematically

$$MMSD=V_{2}R+\frac{V_{2}^{2}}{2d_{2}}$$ (9)

where *V*_2_ is the speed of the following vehicle, *R* represents the PRT of the following driver, *d*_2_ denotes the maximum acceptable deceleration rate taken by the following vehicle. Then, the modified PSD (MPSD) can be expressed as:

$$MPSD=\frac{RD}{MMSD}=\frac{V_{2}\cdot TTC}{V_{2}\cdot R+\frac{V_{2}^{2}}{2d_{2}}}=\frac{TTC}{R+\frac{V_{2}}{2d_{2}}}$$ (10)

MPSD is believed to be more realistic compared to the traditional PSD. However, it is impossible to get the scenario-specific PRT during a survey. In this regard, the distribution of PRT is introduced. According to the previous research, the distribution of PRT is observed to be lognormally distributed [45–48]. Green [48] suggests the log-normal distributions as a mean of 1.3 seconds and a standard deviation of 0.6 second for the crossing and lane change situations. For rear-end situation, PRT is reported by Triggs and Harris [45] to follow a lognormal distribution with a mean of 0.92 second and a standard deviation of 0.28 second. Without loss of generality, we use the lognormal distribution with a mean of 0.92 second and a standard deviation of 0.28 second as the PRT distribution in this study for rear-end situation.

## Validation of the Micro-Traffic Simulation Model

VISSIM is a useful micro-traffic simulation tool, which has been widely used in traffic simulation [40–44, 49]. In this research, VISSIM is applied to simulate the traffic of the investigated section on the Pacific Motorway. To ensure the accuracy of VISSIM on simulation, we validate our simulation model by comparing the speeds and volumes [50–53]. All field data are collected on the investigated section which is located between the exits #20 and #9 of the northbound of Pacific Motorway.

Based on previous studies [26, 37, 54], we use four error tests to assess the differences between the simulation results and the field data: (1) Theil’s inequality coefficient (U); (2) root mean square percentage error (RMSPE); (3) root mean square error (RMSE); (4) mean percentage error (MPE), mathematically,

$$U=\frac{\sqrt{\frac{1}{N_{0}}\sum_{n=1}^{N_{0}}{\left(y_{n}^{s}-y_{n}^{0}\right)}^{2}}}{\sqrt{\frac{1}{N_{0}}\sum_{n=1}^{N_{0}}{\left(y_{n}^{s}\right)}^{2}}+\sqrt{\frac{1}{N_{0}}\sum_{n=1}^{N_{0}}{\left(y_{n}^{0}\right)}^{2}}}$$ (11)

$$RMSPE=\sqrt{\frac{1}{N_{0}}\sum_{n=1}^{N_{0}}{\left(\frac{y_{n}^{s}-y_{n}^{0}}{y_{n}^{0}}\right)}^{2}}$$ (12)

$$RMSE=\sqrt{\frac{1}{N_{0}}\sum_{n=1}^{N_{0}}{\left(y_{n}^{s}-y_{n}^{0}\right)}^{2}}$$ (13)

$$MPE=\frac{1}{N_{0}}\sum_{n=1}^{N_{0}}\left(\frac{y_{n}^{s}-y_{n}^{0}}{y_{n}^{0}}\right)$$ (14)

where $y_{n}^{s}$ represents the simulation value (speed) of the *n* ^*th*^ vehicle in the VISSIM model; $y_{n}^{0}$ denotes the field value of the *n* ^*th*^ vehicle; *N*_0_ is the number of observations. We randomly select 20 vehicles from the field data and record their times. Then these vehicles will be matched with those generated from our simulation model according to the recorded time. In this study, the four error tests are carried out for comparing the speeds of randomly selected vehicles. Five groups of field data are randomly extracted, each of which contains 20 vehicles. The average error tests are aggregated in Table 1. As can be seen in the table, the simulation model performs well.

**Table 1:** Error tests of speeds.

Number of group	RMSE(m/s)	RMSPE (%)	MPE (%)	U (%)
1	2.88	11.31	5.75	0.24
2	2.77	11.30	4.13	0.23
3	3.39	13.35	7.42	0.28
4	3.36	12.34	6.67	0.26
5	2.99	11.74	5.64	0.24
Average	3.08	12.01	5.92	0.25

We further use Geoffery E. Heavers (GEH) test to conduct volume validation. Geoffery E. Heavers (GEH), a modified chi-square statistics, has been widely employed to compare the fitness between simulation and field data [51–53]. Mathematically, GEH can be represented as

$$GEH=\sqrt{\frac{{\left(S-F\right)}^{2}}{(S+F)/2}}$$ (15)

where S denotes simulated data, while F represents field data. GEH is regarded as a good statistical measure by considering both relative and absolute differences between simulated and field data. In this study, the field and simulated data of 20 randomly chosen time periods are compared. Each time period contains one hour. Table 2 shows the results on comparison of total flow per hour between field and simulated data. As suggested by Dowling et al. [51] and Holm et al. [52], a GEH value for sum volume of all links less than 4 is considered as a good fit. Therefore, Table 2 further demonstrates the effectiveness of the simulation model.

**Table 2:** Comparison of total flow per hour between field and simulated data.

Number of time period	Field data	Simulated data	GEH
1	6045	5966	1.02
2	5823	5752	0.93
3	5088	5019	0.97
4	4801	4732	1.00
5	4547	4504	0.64
6	4175	4155	0.31
7	5770	5691	1.04
8	5992	5859	1.73
9	5995	5869	1.64
10	4888	4832	0.80
11	5150	5086	0.89
12	2747	2739	0.15
13	4424	4383	0.62
14	3918	3873	0.72
15	4552	4499	0.79
16	4491	4439	0.78
17	5779	5735	0.58
18	6011	5901	1.43
19	6024	5923	1.31
20	4866	4798	0.98

## Performance Analysis of Modified Surrogate Indicators

### Data Description

In order to test the performances of the traditional and modified surrogate indicators, we carry out a case study on the Pacific Motorway. The Pacific Motorway (M1) in Queensland, Australia, is the major urban road corridor connecting Tugun to the Sunshine Coast hinterland via the Gold Coast and Brisbane. In this research, the investigated section is chosen between the exits #20 and #9 of the northbound Pacific Motorway. Based on the traffic data provided by the department of TMR in Queensland, the average traffic volume, speed and time headway for each 15-minute from 21^st^ July 2014 (Monday) to 27^th^ July 2014 (Sunday) are available for each lane, respectively. By setting these parameters in VISSIM, the traffic condition of the investigated section can be simulated. Thus the risk of the whole section can be evaluated by different surrogate indicators based on the trajectories generated by VISSIM. Furthermore, according to the historical crash data provided by TMR, all rear-end crashes for this section from 2005 to 2013 are considered into analysis.

### A Comparative Study of Crash Surrogate Indicators

With the aim of testing the impact of the PRT, we compare the performances of traditional and modified surrogate indicators on the crash prediction. In this study, we use VISSIM to simulate the traffic situation of investigated section for 168 successive time periods (24 hours per day times 7 days) from Monday to Sunday. Based on the trajectory data, we exacted the traffic data such as the speed of leading (*V*_1_) and following vehicle (*V*_2_), the length of the leading vehicle (*l*_1_), the time headway of the following vehicle (*h*_2_) in any car-following scenario. Suggested by Vogel [55], the gap distance of this car following scenario (*D*_1−2_) can be estimated as (*V*_2_ × *h*_2_ − *l*_1_). According the definitions of surrogates, the risk can be represented by different surrogate indicators. By considering the randomness or heterogeneity of PRT and MADR, we use Monte-Carlo method to calculate the risk by applying different surrogates. For each car-following scenario, the risk is calculated based on 1000 seeds for both distributions. The concepts of individual and societal risk were proposed by Considine [56] and have been widely used in safety evaluation [26, 30, 57, 58]. Individual risk is defined as the crash risk or threat to an individual motorist, which is regarded as the likelihood of collision occurring to the individual traveler *i*. For each car-following scenario, the individual risk can be obtained by comparing the surrogate value and the surrogate threshold. In this study, the thresholds of DRAC (MDRAC), CPI (MCPI) and PSD (MPSD) are 3.4 m/s^2^, 0 and 1 respectively. Further, the societal risk is defined as the combined risk of all individual risks to all of the affected motorists during time period *T* measured by surrogate indicator *j*, mathematically represented by

$$SR_{j}=\sum_{i=1}^{M}\int_{0}^{T}IR_{ij}(t)dt\approx \sum_{i=1}^{M}\sum_{t=0}^{N}IR_{ij}(t)\cdot \tau_{sc}$$ (16)

where *IR*_*ij*_(*t*) represents the individual risk of the discrete scenario *i* at discrete time *t* measured by surrogate *j*, *τ*_*sc*_ is the time-scan interval, there are a total of *N* time instances during time period *T*. In this study, the probabilistic properties of crashes during weekdays are found to be different with those during weekends. In this regard, we categorize all the data into weekdays and weekends for better representation.

**Fig 2:**
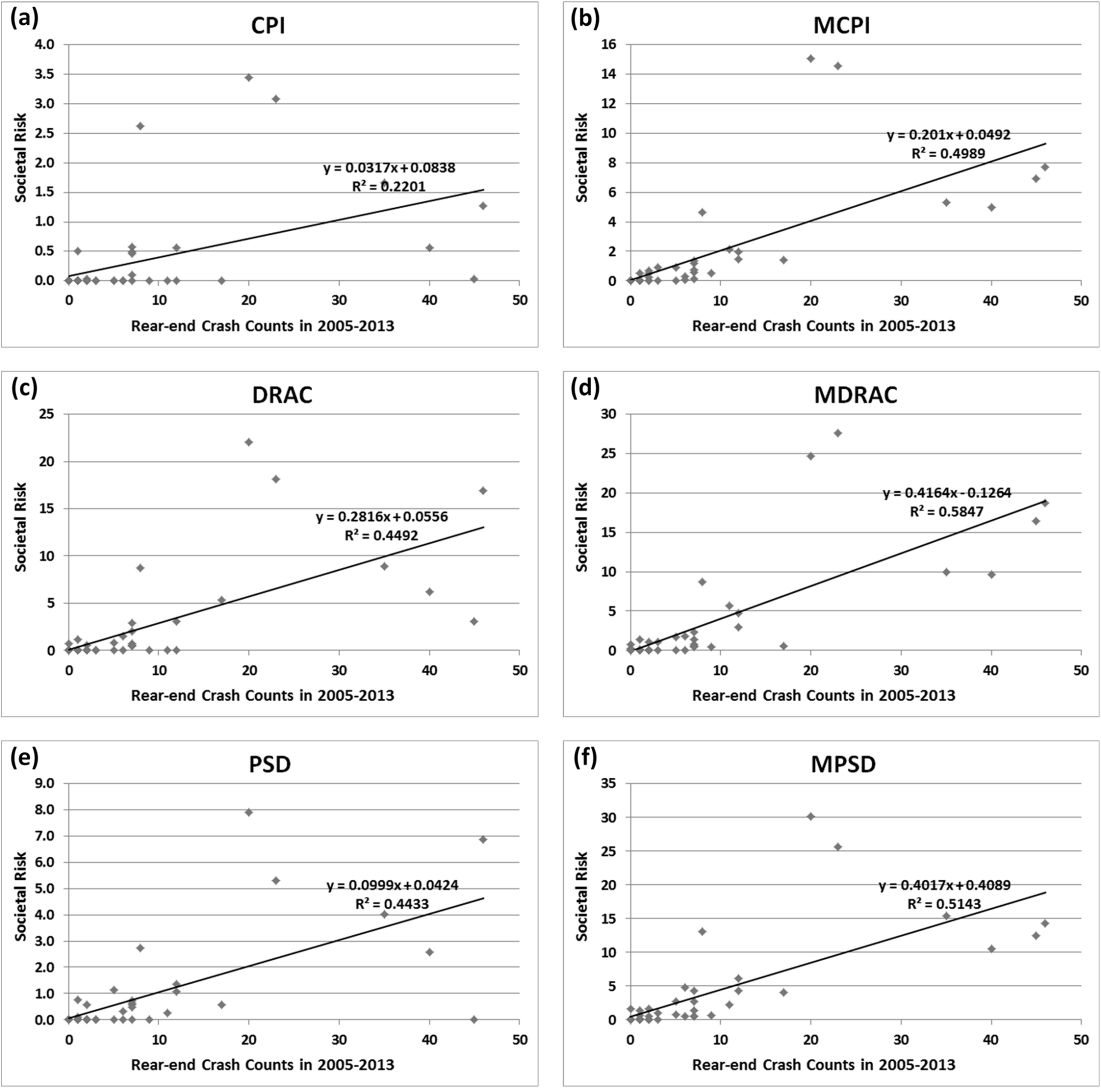
The relationship between societal risk and crash counts.

Fig 2 shows the linear relationships between the crash counts and societal risks represented by different surrogate indicators (P values<0.05). The R square value indicates how well the societal risk fits crash counts in a linear model. The higher R square value indicates better performance of the surrogate indicator on predicting crash in a linear relationship. As can be seen in Fig 2, the R squares of modified surrogate indicators MDRAC (0.5847), MCPI (0.4989) and MPSD (0.5143) are higher than those of the traditional surrogates DRAC (0.4492), CPI (0.2201) and PSD (0.4433), respectively. Besides, it is found that the R square difference (0.2788) between MCPI and CPI is greater than that (0.0710) between MPSD and PSD and that (0.1355) between MDRAC and DRAC.

## Findings and Discussions

According to our analysis, it is found that the modified surrogate indicators have higher R squares compared with traditional ones. There is no surprise that the crash prediction performance is improved by considering the PRT. Further, the impact on CPIs is much more significant than that on the other two indicators. The possible reason relies on their different methodology of crash mechanism. By considering the PRT in the crash mechanism, MDRAC will be measured by TTC, PRT and speed difference. For the cases in which PRT is greater than TTC, a collision will happen before the following driver reacts to stop, thus the MDRAC is infinity and the value of MCPI is 1. Accordingly, in those cases, the value of CPI can be greatly changed by taking into account the PRT. Consequently, PRT is a critical parameter to determine potential risk in the crash mechanism of MCPI. However, for those cases in which PRT is greater than TTC, the values of DRAC are likely to be greater than 3.4 m/s^2^ due to the small TTC. Then these scenarios are considered as dangerous. Thus the R square difference between DRAC and MDRAC is not as big as that of CPI and MCPI. In other words, the consideration of the PRT affects the performance of MCPI in a higher degree than that of MDRAC. Besides, in the crash mechanism of PSD, PRT is only adding to the MSD which can slightly change the ratio of RD and MSD, hence the consideration of the PRT would just slightly decrease MPSD.

It is of great importance to carry out more studies by considering the PRT into the surrogate modelling. Two further works can be done based on this study. Firstly, the PRT can be incorporated into the crash mechanisms of surrogate indicators which are designed for the crossing and lane changing situations. Secondly, another comparative study can be accomplished to examine the different impacts of PRT on surrogate indicators in terms of different speed limits.

## Abbreviations

*CPIi*: CPI value for *i* ^th^ car-following scenario
*d2*: Deceleration rate of following vehicle
*D1–2*: Distance gap between the leading and following vehicle
*DRAC*: Decelerate rate to avoid a crash
*DRACi(t)*: DRAC value for *i* ^th^ car-following scenario at discrete time *t*
*GEH*: Result of Geoffery E. Heavers (GEH) test
*F*: Field data
*IRij*: Individual risk of the discrete scenario *i* measured by surrogate *j*
*MADRi*: MADR value for *i* ^th^ car-following scenario
*MCPIi*: Modified CPI value for *i* ^th^ car-following scenario
*MDRAC*: Modified decelerate rate to avoid a crash
*MDRACi(t)*: Modified DRAC value for *i* ^th^ car-following scenario at discrete time *t*
*MMSD*: Modified minimum stopping distance required
*MPE*: Mean percentage error
*MPSD*: Modified proportion of stopping distance
*MSD*: Minimum stopping distance required
*N0*: Number of observations
*PSD*: Proportion of stopping distance
*R*: Reaction time of the following vehicle
*RD*: Distance between the initial point and the potential point of collision
*RMSE*: Root mean square error
*RMSPE*: Root mean square percentage error
*S*: Simulation data
*SRj*: Societal risk of surrogate j
*T*: Total time duration inspected
*TTC*: Time to collision
*U*: Theil’s inequality coefficient
*V1*: Speed of the leading vehicle
*V2*: Speed of the following vehicle
$y_{n}^{s}$: Simulation value of the *n* ^*th*^ vehicle in the VISSIM model
$y_{n}^{0}$: Field value of the *n* ^*th*^ vehicle
*τsc*: Time-scan interval
*Δt*: Time duration of time interval
